# A WHO-led global strategy to control greenhouse gas emissions: a call for action

**DOI:** 10.1186/s12992-023-01008-6

**Published:** 2024-01-02

**Authors:** Matthew F Chersich, Nicholas Brink, Marlies H Craig, Gloria Maimela, Fiona Scorgie, Stanley Luchters

**Affiliations:** 1 Wits RHI, Faculty of Health Sciences, University of the Witwatersrand, Johannesburg, South Africa; 2 Centre for Sexual Health and HIV/AIDS Research (CeSHHAR), Harare, Zimbabwe

**Keywords:** Climate and health, Greenhouse gases, Hazardous substances, Public health, Emissions control, Policy

## Abstract

**Background:**

Climate change, driven by anthropogenic greenhouse gas emissions, is among the greatest threats to human health. The World Health Organisation (WHO), has led global efforts to respond to emerging public health threats including the control of hazardous substances such as tobacco, alcohol, lead and asbestos, with remarkable health gains.

**Body:**

Despite WHO’s clear messaging on the enormous and growing health risks of climate change, greenhouse gases are not yet classified as hazardous substances, requiring control through a global strategy or framework. Additionally, WHO has not classified disease attributable to climate change as a result of the promulgation of these hazards as a Public Health Emergency of International Concern (PHEIC), despite the serious and preventable health risks it poses globally. Several historical precedents set the stage for WHO to declare excess greenhouse gases as health hazards, including the control of ozone-depleting substances and breast-milk substitutes where the public benefit of control exceeded the potential benefit of their promulgation. In addition, WHO’s undertaking within the International Health Regulations to protect global health, providing imperative to declare climate change a PHEIC, with Tedros Adhanom Ghebreyesus, director-general of WHO, declaring: “The climate crisis is a health crisis, fuelling outbreaks, contributing to higher rates of noncommunicable diseases, and threatening to overwhelm our health workforce and health infrastructure”. Importantly, the health sector, perhaps more than other sectors, has successfully overcome formidable, vested interests in combatting these threats to health.

**Conclusion:**

It is thus imperative that WHO make full use of their credibility and influence to establish a global framework for the control of greenhouse gases through the declaration of excess greenhouse gas emissions as a hazardous substance, and declaring climate change a PHEIC. Who else is better placed to drive the considerable societal transformation needed to secure a liveable future?

**Supplementary Information:**

The online version contains supplementary material available at 10.1186/s12992-023-01008-6.

## Background

Climate change, driven by anthropogenic greenhouse gas emissions [1] is among the greatest threats to human health [2, 3]. WHO, since its founding, has led global efforts to respond to emerging public health threats. It was instrumental in the control of hazardous substances such as tobacco [4], alcohol [5], lead [6, 7]and asbestos [8] with remarkable health gains [9]. In addition, through the implementation of the International Health Regulations [10], it has been critical in the coordinated response to public health emergencies, most recently the COVID-19 pandemic [11, 12].

## Main text

Although WHO is clear about the enormous and growing health risks of climate change, greenhouse gases are not yet classified as hazardous substances, requiring control through a global strategy or framework [13]. Additionally, WHO has not classified climate change as a result of the promulgation of these hazards as a Public Health Emergency of International Concern (PHEIC), despite the serious and preventable health risks it poses globally.

It is thus very encouraging that a recent article by Campbell-Lendrum and co-authors from WHO mention future actions to reduce carbon emissions as one of three ‘grand challenges’ in the health sector’s response to climate change [14]. The authors note that such actions may include classic public health measures such as behaviour change communication around high-emission practices, the application of carbon emission estimates on product labels, marketing restrictions, and pricing mechanisms on high-emitting substances, where healthcare professionals serve as a trusted voice in this discourse. Calls from the director-general of WHO, Tedros Adhanom Ghebreyesus, for urgent climate action must be commended, declaring: “The climate crisis is a health crisis, fuelling outbreaks, contributing to higher rates of noncommunicable diseases, and threatening to overwhelm our health workforce and health infrastructure” [15]. Clearly, addressing the crisis requires an unprecedented coordinated global response and vigorous measures to counter the formidable fossil-fuel lobby [16].

Several historical precedents set the stage for WHO to declare excess greenhouse gases (principally carbon dioxide and methane) as health hazards, and to declare disease attributable to climate change a PHEIC. These actions would set the stage for a coordinated health sector approach to emission control. Firstly, the example of ozone-depleting substances being phased-out to protect the ozone layer under the auspices of the Montreal protocol marked an instance where chemicals causing indirect health harms through atmospheric changes were regulated [17], and the guidance on the use of the IHR clearly stating *“chemical contamination of… the environment****”*** as a potential public health risk [18]. Listing air pollution as a chemical of major public health concern also illustrates WHO’s commitment to curbing harmful environmental hazards caused by anthropogenic activities [13]. Secondly, WHO’s declaration of COVID-19 as a PHEIC, resulted in a globally coordinated response, mobilising vital government and private resources despite significant disruptions to travel and trade in order to protect global health [11]. These actions allowed us to return to our coveted freedoms once these threats had been sufficiently prevented and mitigated.

Thirdly, the experience with control of breastmilk substitutes showed that even though a product may have high utility and even health benefits, it’s propensity for harm necessitated restrictions [19]. By analogy, at present, many social and health functions rely on products, processes and services that emit greenhouse gases, but such benefits need to be balanced against the overall danger posed by their emissions. In many instances high-emission services can be replaced with low-emission alternatives, or where unavoidable, can be reduced to a minimum, through higher efficiency [1]. The COVID-19 pandemic response illustrated quite clearly that short-term pain was necessary for long-term gain when responding to public health emergencies [12].

Importantly, the health sector, perhaps more than other sectors, has successfully overcome formidable, vested interests, such as privately funded lobby groups against the control of tobacco [20] and, at times, governments such as the Reagan administration’s resistance to control of breastmilk substitutes [21]. Indeed, the stark power differentials between the fossil fuel industry and affected populations makes it even more important that organizations like WHO make full use of their credibility and influence in the interests of safeguarding global health security.

Finding novel ways to protect health during the anthropogenic era is one of the most important challenges the health sector has yet faced. We call on all *National IHR Focal Points*, specifically those at highest risk of climate attributable disease burden, to consider submitting a notification to the WHO of a potential PHEIC. This call is in keeping with the expanded scope of the 2005 revision of the IHR, and is intended not as an open-ended declaration, but rather as an acknowledgement of the crisis we face, and a mechanism through which to provide a globally-coordinated response to reduce greenhouse gas emissions in the interest of safeguarding public health.

Should the IHR fail to provide such a mechanism, we call for an amendment to these regulations, or the urgent establishment of a global framework or convention for the control of greenhouse gas emissions. Is it time to consider, for example, health warning labels and graphic imagery on high-emission products to communicate the impacts of climate change? Perhaps emissions labelling on food and other products may help quantify, and lessen, our individual contribution to climate change, by encouraging behavioural changes targeting high-emission diets and wasteful food practices, for example. Taxation of high-emission products at levels commensurate with their health harms, would provide a critical source of funding for a ‘Global Fund for Planetary Health’ type of mechanism.

## Conclusion

In summary, the role of the health sector in the climate change response extends far beyond supporting adaptation. The health sector has a critical window of opportunity to support the control of excessive, and still growing, greenhouse gas emissions, through a WHO-led mechanism and by declaring disease attributable to climate change a PHEIC. Who else is better placed to drive the considerable societal transformation needed to secure a liveable future?

### Electronic supplementary material

Below is the link to the electronic supplementary material.

Supplementary Material 1

Supplementary Material 2

## Data Availability

Not applicable.

## Abbreviations

COVID-19: Coronavirus Disease 2019
IHR: International Health Regulations
WHO: World Health Organisation
PHEIC: Public Health Emergency of International Concern
