# Supplementary material for: A WHO-led global strategy to control greenhouse gas emissions: a call for action
Source: Global Health. 2024 Jan 2;20:4. doi: 10.1186/s12992-023-01008-6 (PMC10759590; doi:10.1186/s12992-023-01008-6)
Supplement: Supplementary file 1 — Supplementary Material 1 [file 12992_2023_1008_MOESM1_ESM.pdf]

ANNEX 2

DECISION INSTRUMENT FOR THE ASSESSMENT AND NOTIFICATION OF EVENTS THAT MAY CONSTITUTE A PUBLIC HEALTH EMERGENCY OF INTERNATIONAL CONCERN

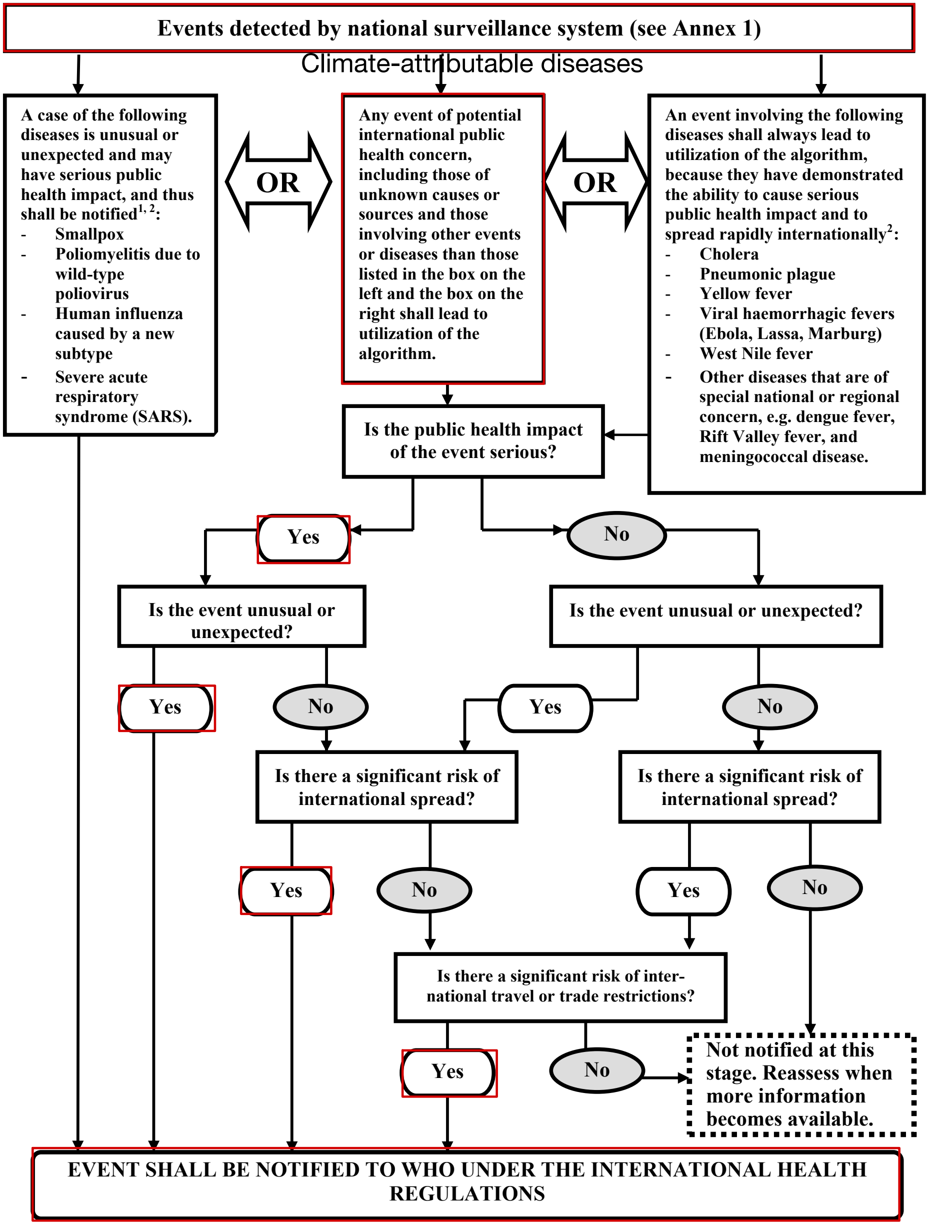

[1] As per WHO case definitions.
[2] The disease list shall be used only for the purposes of these Regulations.
