# Supplementary material for: A WHO-led global strategy to control greenhouse gas emissions: a call for action
Source: Global Health. 2024 Jan 2;20:4. doi: 10.1186/s12992-023-01008-6 (PMC10759590; doi:10.1186/s12992-023-01008-6)
Supplement: Supplementary file 2 — Supplementary Material 2 [file 12992_2023_1008_MOESM2_ESM.pdf]

EXAMPLES FOR THE APPLICATION OF THE DECISION INSTRUMENT FOR THE ASSESSMENT AND NOTIFICATION OF EVENTS THAT MAY CONSTITUTE A PUBLIC HEALTH EMERGENCY OF INTERNATIONAL CONCERN

The examples appearing in this Annex are not binding and are for indicative guidance purposes to assist in the interpretation of the decision instrument criteria.

DOES THE EVENT MEET AT LEAST TWO OF THE FOLLOWING CRITERIA?

Is the public health impact of the event serious?

I. Is the public health impact of the event serious?

1. *Is the number of cases and/or number of deaths for this type of event large for the given place, time or population?*

2. *Has the event the potential to have a high public health impact?*

THE FOLLOWING ARE EXAMPLES OF CIRCUMSTANCES THAT CONTRIBUTE TO HIGH PUBLIC HEALTH IMPACT:

- ✓ Event caused by a pathogen with high potential to cause epidemic (infectiousness of the agent, high case fatality, multiple transmission routes or healthy carrier).
- ✓ Indication of treatment failure (new or emerging antibiotic resistance, vaccine failure, antidote resistance or failure).
- ✓ Event represents a significant public health risk even if no or very few human cases have yet been identified.
- ✓ Cases reported among health staff.
- ✓ The population at risk is especially vulnerable (refugees, low level of immunization, children, elderly, low immunity, undernourished, etc.).
- ✓ Concomitant factors that may hinder or delay the public health response (natural catastrophes, armed conflicts, unfavourable weather conditions, multiple foci in the State Party).
- ✓ Event in an area with high population density.
- ✓ Spread of toxic, infectious or otherwise hazardous materials that may be occurring naturally or otherwise that has contaminated or has the potential to contaminate a population and/or a large geographical area.

3. *Is external assistance needed to detect, investigate, respond and control the current event, or prevent new cases?*

THE FOLLOWING ARE EXAMPLES OF WHEN ASSISTANCE MAY BE REQUIRED:

- ✓ Inadequate human, financial, material or technical resources – in particular:
  - – insufficient laboratory or epidemiological capacity to investigate the event (equipment, personnel, financial resources);
  - – insufficient antidotes, drugs and/or vaccine and/or protective equipment, decontamination equipment, or supportive equipment to cover estimated needs;
  - – existing surveillance system is inadequate to detect new cases in a timely manner.

IS THE PUBLIC HEALTH IMPACT OF THE EVENT SERIOUS?

Answer "yes" if you have answered "yes" to questions 1, 2 or 3 above.

Is the event unusual or unexpected?

II. Is the event unusual or unexpected?

4. *Is the event unusual?* Anthropogenic climate change significant deviation from natural variability

THE FOLLOWING ARE EXAMPLES OF UNUSUAL EVENTS:

- ✓ The event is caused by an unknown agent or the source, vehicle, route of transmission is unusual or unknown.
- ✓ Evolution of cases more severe than expected (including morbidity or case-fatality) or with unusual symptoms.
- ✓ Occurrence of the event itself unusual for the area, season or population.

5. *Is the event unexpected from a public health perspective?*

THE FOLLOWING ARE EXAMPLES OF UNEXPECTED EVENTS:

- ✓ Event caused by a disease/agent that had already been eliminated or eradicated from the State Party or not previously reported.

IS THE EVENT UNUSUAL OR UNEXPECTED?

Answer "yes" if you have answered "yes" to questions 4 or 5 above.

Is there a significant risk of international spread?

III. Is there a significant risk of international spread?

6. *Is there evidence of an epidemiological link to similar events in other States?*

7. *Is there any factor that should alert us to the potential for cross border movement of the agent, vehicle or host?*

THE FOLLOWING ARE EXAMPLES OF CIRCUMSTANCES THAT MAY PREDISPOSE TO INTERNATIONAL SPREAD:

- ✓ Where there is evidence of local spread, an index case (or other linked cases) with a history within the previous month of:
  - international travel (or time equivalent to the incubation period if the pathogen is known);
  - participation in an international gathering (pilgrimage, sports event, conference, etc.);
  - close contact with an international traveller or a highly mobile population.
- ✓ Event caused by an environmental contamination that has the potential to spread across international borders.
- ✓ Event in an area of intense international traffic with limited capacity for sanitary control or environmental detection or decontamination.

IS THERE A SIGNIFICANT RISK OF INTERNATIONAL SPREAD?

Answer "yes" if you have answered "yes" to questions 6 or 7 above.

Risk of international restrictions?	**IV. Is there a significant risk of international travel or trade restrictions?**
	8. *Have similar events in the past resulted in international restriction on trade and/or travel?* Consider previous PHEIC such as COVID-19 as well as previous environmental catastrophes such as Fukushima
	9. *Is the source suspected or known to be a food product, water or any other goods that might be contaminated that has been exported/imported to/from other States*?
	10. *Has the event occurred in association with an international gathering or in an area of intense international tourism?*
	11. *Has the event caused requests for more information by foreign officials or international media?*
	**IS THERE A SIGNIFICANT RISK OF INTERNATIONAL TRADE OR TRAVEL RESTRICTIONS?**<br><br>**Answer "yes" if you have answered "yes" to questions 8, 9, 10 or 11 above.**

States Parties that answer "yes" to the question whether the event meets any two of the four criteria (I-IV) above, shall notify WHO under Article 6 of the International Health Regulations.
